# Supervision as a tool for building surgical capacity of district hospitals: the case of Zambia

**DOI:** 10.1186/s12960-020-00467-x

**Published:** 2020-03-26

**Authors:** Jakub Gajewski, Nasser Monzer, Chiara Pittalis, Leon Bijlmakers, Mweene Cheelo, John Kachimba, Ruairi Brugha

**Affiliations:** 1 Institute of Global Surgery, Royal College of Surgeons in Ireland, 123 St Stephen’s Green, Dublin 2, Ireland; 2 Division of Population Health Sciences, Royal College of Surgeons in Ireland, 123 St Stephen’s Green, Dublin 2, Ireland; 3 Radboud University Medical Centre Netherlands, Geert Grooteplein Zuid 10, 6525 Nijmegen, GA Netherlands; 4 Surgical Society of Zambia, Department of Surgery, University Teaching Hospital, P.O. Box 50110, Lusaka, Zambia

**Keywords:** Global surgery, Task shifting, Non-physician clinicians, Zambia, Medical licentiates

## Abstract

**Introduction:**

Many countries in sub-Saharan Africa have adopted task shifting of surgical responsibilities to non-physician clinicians (NPCs) as a solution to address workforce shortages. There is resistance to delegating surgical procedures to NPCs due to concerns about their surgical skills and lack of supervision systems to ensure safety and quality of care provided. This study aimed to explore the effects of a new supervision model implemented in Zambia to improve the delivery of health services by surgical NPCs working at district hospitals.

**Methods:**

Twenty-eight semi-structured interviews were conducted with NPCs and medical doctors at nine district hospitals and with the surgical specialists who provided in-person and remote supervision over an average period of 15 months. Data were analysed using ‘top-down’ and ‘bottom-up’ thematic coding.

**Results:**

Interviewees reported an improvement in the surgical skills and confidence of NPCs, as well as better teamwork. At the facility level, supervision led to an increase in the volume and range of surgical procedures done and helped to reduce unnecessary surgical referrals. The supervision also improved communication links by facilitating the establishment of a remote consultation network, which enabled specialists to provide real-time support to district NPCs in how to undertake particular surgical procedures and expert guidance on referral decisions. Despite these benefits, shortages of operating theatre support staff, lack of equipment and unreliable power supply impeded maximum utilisation of supervision.

**Conclusion:**

This supervision model demonstrated the additional role that specialist surgeons can play, bringing their expertise to rural populations, where such surgical competence would otherwise be unobtainable. Further research is needed to establish the cost-effectiveness of the supervision model; the opportunity costs from surgical specialists being away from referral hospitals, providing supervision in districts; and the steps needed for regular district surgical supervision to become part of sustainable national programmes.

## Background

Many countries in sub-Saharan Africa (SSA) have adopted task shifting of surgical responsibilities to non-physician clinicians (NPCs) as a solution to address current shortages in the specialised surgical workforce [1, 2]. NPCs are often the main or only cadre of clinicians working in rural district-level hospitals (DLHs); as such, they play a critical role in the delivery of first-line essential surgical services to underserved rural populations. Studies have demonstrated the efficacy of surgical task shifting to NPCs [3–5] due to the lower cost and shorter duration of their training compared to other cadres [6], as well as good retention rates in rural facilities [4, 7, 8]. However, there is still some resistance to delegating surgical procedures to NPCs [3, 7, 9] as they lack the qualifications, training and status of physicians, sometimes making them reluctant to undertake certain procedures, even if they are trained to perform them [10, 11]. As suggested by recent studies, one way to support surgically active NPCs is through continuing education and in-service training, including programmes of regular visits by specialists to facilitate skills transfer through practical learning [9, 12, 13]. Supervision allows specialists to monitor the surgical performance of district hospital-level NPCs, while offering opportunities to further develop NPCs surgical skills in a safe and controlled environment [6, 9, 14–16]. This may contribute to reducing risks and ensuring overall quality of care in DLHs [6, 9, 14–16].

Zambia is a prime example of a SSA country battling a shortage in healthcare workers [17], particularly in DLHs. With almost two thirds of its population in rural areas [18], Zambia continues to rely heavily on their NPCs, locally known as medical licentiates (MLs), for the provision of surgical care particularly in its rural hospitals. In the National Health Sector Strategic Plan 2011–2015 [19], the Ministry of Health (MoH) recognised among its key priorities the need to improve skills levels for existing health staff, including the MLs, through new in-service training models. Undertaking regular in-service training is also part of the official ML job description.

### The supervision model

The Clinical Officer Surgical Training in Africa (COST-Africa) project, implemented in Zambia in 2011–2016 [10], tested such a model through an intervention involving two elements: firstly, an intensive, 3-month course in surgery for practicing MLs, already trained in basic general surgery, and secondly, a programme of quarterly onsite supervisory visits by general surgeons to oversee the COST-Africa MLs once deployed to DLHs. Each of the provincial hospitals that managed surgical referrals for the selected DLHs included a lead specialist surgeon who was recruited to be a surgeon supervisor. Incentives for supervisors were agreed in line with local per diem rates for outreach. The DLHs’ 2-day supervisory visits comprised the following activities: meeting with the hospital management; meeting with MLs and other surgical staff; review and advice on theatre management and operational procedures; review of surgical data to assess performance, quality and outcomes; hands-on training on the operating theatre (OT); supervision of ward rounds; and surgical patient reviews. All supervisors adhered to the agreed schedule of visits and completed planned visits. After each visit, supervising surgeons filled in a trip report summarising the activities conducted at the visits and indicating areas for improvements. The supervision continued for 15 months on average in each of the facilities.

The first part of the intervention (training outcomes) was evaluated through a randomised control trial (RCT); findings yielded interesting insights into the benefits of using surgically trained MLs in the provision of surgical care for rural populations, including a shift in surgical care (task shifting) from doctors to MLs and increases in caesarean section (C/S) rates [20]. The evaluation reported in the previous publication did not assess the supervision model. This paper examines the delivery of capacity building through supervision. It captures the supervisors’ and supervisees’ experiences and aims to provide lessons on the surgical supervision model at individual, facility and system levels. The model and its evaluation, which are reported in this paper, contributed an important component to Zambia’s National Surgical Obstetric and Anaesthesia Plan (NSOAP) [21]. This paper was guided by the following questions: (1) how did the supervisory visits affect surgical care in Zambian rural hospitals? and (2) what were the enablers and impediments to effective supervision?

## Methods

The methods are reported according to the EQUATOR Network’ COREQ checklist [22] and the Standards for Reporting Qualitative Research (SRQR) [23]; however, some of the checklist items were merged to reflect the conduct of the study. We conducted a qualitative study of the surgical supervision model implemented by COST-Africa in Zambian rural district hospitals, using semi-structured interviews with MLs, district medical officers (DMOs) and supervising surgeons to examine the experiences of participants and to explore the effectiveness and feasibility of the model. Two experienced qualitative researchers with a background in social sciences and formal training in qualitative interviewing conducted collected the data. Both were employed by the project and involved in the implementation of the COST-Africa intervention allowing them to gain good knowledge about the context of the study. One researcher was of Zambian nationality and one was foreign, but with a number of years of experience working in Zambia. This was also believed to have a positive effect on the desired depth of the interviews. Both had previously developed relationships with most of the study participants from interactions through the project activities.

### Sampling

The study sample consisted of all eligible MLs (12) and DMOs (11) from nine district hospitals, as well as five supervising surgeons from central hospitals. Interviewed surgeons were responsible for the supervision of the MLs during the COST-Africa project. Interviewed MLs were directly involved in the COST-Africa project at hospitals included in the study. DMOs included in the sample were in charge of the hospitals involved in the COST-Africa intervention and interacted both with the MLs and their supervisors, providing insights into the possibilities for roll-out of the intervention. Data saturation was not a consideration, because the intention was to interview all study participants (MLs) and some of their colleagues working in the intervention hospitals.

### Data collection, context and ethics

Topic guides specific for each interviewed cadre were developed by the project researchers following project interactions with them and a review of published literature. All interviews were conducted towards the latter stages of the project (June to August 2016). The interviews, which lasted approximately 1 h each, were performed face-to-face during hospital site visits or as part of meetings related to COST-Africa activities. Informed consent was obtained verbally prior to each interview; the researchers explained to the respondents the purpose of the study and the type of questions being asked, and ensured the confidentiality and anonymity of each participant. The study was reviewed and approved by the University of Zambia Biomedical Research Ethics Committee (ref: 018–0312) and the Research Ethics Committee of the Royal College of Surgeons in Ireland (ref: REC727).

### Data processing and analysis

Interviews were audio-recorded and subsequently transcribed using MS Word by one team researcher (CM). Using a combination of ‘top-down’ and ‘bottom-up’ thematic coding [24], two members of the study team (NM, JG) designed a coding frame with 16 initial codes. The codes were derived from the literature review, previous studies done by the team and new areas emerging from the data analysis of the interviews conducted for this study. Thematic codes were discussed with the remainder of the study team and filtered based on their relevance to the main research question leading to the final 10 codes applied in the process of data analysis. The presentation of results in this manuscript reflects the final thematic framework focusing on identifying the benefits of the supervision model and obstacles to its full utilisations.

## Results

### Benefits of surgical supervision

The supervision resulted in a chain of interlinked effects at individual, hospital and health system levels. The interviews revealed that in most instances, the MLs had never had a chance to operate under the supervision of surgical specialists outside of their earlier ML training programme. Respondents described the in-service supervision as ‘a privilege’, appreciating the opportunity to interact and operate together with some of the most senior specialist surgeons in the country, unavailable to them otherwise. In some hospitals, participants noted that the whole theatre team had ‘great respect’ for the supervisor and that clinicians who normally do not work in the surgical department would come to learn from him. Most interviewed MLs valued the opportunity to apply in practice the skills learned in class, by performing operations under the supervision of the visiting specialist. This allowed MLs to improve their practical skills and increase their confidence. As explained by one of the respondents:

For me before COST-Africa I had no confidence to carry out certain procedures. The support we received from Dr. X (*the supervising specialist*) was very helpful for me. It really built up my confidence in surgery. (ML 1)

Supervising surgeons stated that providing training directly at the facility where local clinicians practise ensures optimal utilisation of available staff and resources. This also allows to work within the limitations of each facility in terms of availability of infrastructure, equipment and supplies, and enables the DLH staff to develop local solutions.

As well as gaining confidence by having a dedicated supervisor working side by side with them, several MLs reported the acquisition of new skills and ability to do cases that they ‘have never done in the past’. With the guidance of the supervising surgeons, they expanded their field of competence and carried out essential surgeries that are technically more challenging than the routine C/S and elective hernias—cases that are expected to be done at the district level. The management of pathologies found during emergency laparotomies was one such area, as described by a respondent:

Yes, those are difficult. So he has given us a lot of skills and then the removal of some of the cysts. There are sometimes big cysts in the abdomen. So he has taught us the skills, how to tackle and approach such. So he has been a good teacher. (ML 5)

The supervision also helped to improve surgical teamwork at the DLHs. In our study, almost all of the interviewed MDs reported undertaking both administrative and clinical roles, while the majority of MLs reported devoting their time almost entirely to surgery. In some facilities, this meant that the MLs and MDs did not have many chances to operate together. Sometimes disputes arose between the two cadres when medical doctors refused to acknowledge the MLs’ advanced skill sets. Critical to the success of the intervention was that the supervising surgeons often resolved these conflicts by acting as higher-level mediating figures, contributing to more cooperative working environments. This was explained by one of the supervising surgeons:

He (*the ML*) was more skilled than them (*the MDs*) so there was a bit of apprehension, some kind of competition, but with time I went there and sat them down, I talked to them and we managed to iron out the friction between the MLs and the local doctors. I think they even established a very good relationship. (SS 4)

At the facility level, the increased confidence and skills of district surgical clinicians due to the supervision reportedly had a positive effect on the volume and range of surgical procedures offered at the hospital, with some cases which previously would have been referred to a higher-level hospital now managed locally. This view was reported by respondents across all interviewed cadres, as described by a supervising specialist:

You could even see that after the supervision, his output (*ML*) actually went up. He could be able to handle most of the cases without having to refer to me (SS 4)

In addition, the supervision resulted in improved communication channels between DLHs and central hospitals. Having established personal links with the supervising surgeons, MLs were able to contact their supervisors directly using their mobile phones when needing guidance about patient management, particularly in the case of more complex surgeries. Both MLs and supervisors considered this communication to be beneficial in supporting decision-making and recalled several examples when the ML performed procedures at the DLH which he had initially wanted to refer. MLs reported that the supervising specialists were able to remotely guide them through a particular surgical procedure so that low-risk patients could be treated locally and referral was avoided. When poor postoperative patient outcomes were anticipated, their advice was to refer without attempting any surgery. This contributed to a better utilisation of district hospital resources.

If you don’t have the knowledge even the simplest thing can be a very complicated thing and very costly. Otherwise imagine if we had no Dr. *X* to consult and contact, the only option was (*to put the patient*) on the ambulance, (*and handle the case as*) an emergency to UTH (*University Teaching Hospital*) […]. And imagine how much it would have cost us, so I have just described the value Dr. *X* has added to the hospital. (ML 9)

In Zambia, DLHs are often affected by shortages of surgical and anaesthetic supplies, and communication channels with higher-level hospitals are limited. Yet, several surgeons in our study reported acting as an advocate on behalf of the district hospitals they visited as a supervisor, supporting them in obtaining surgical supplies when critical shortages hindered surgical service delivery, and more generally raising awareness at higher levels about the needs of DLHs.

I can easily tell the PMO (*Provincial Medical Officer*) because we meet almost on a daily basis. It is easy for me to do things because if I tell him, “Doc there is no ketamine and the theatre was suffering, then he would act almost immediately”. I would go to the pharmacy and they can give us extra or talk to the provincial pharmacist. (SS 1)

Finally, regular surgical supervision contributed towards improving the hospitals’ safety standards by addressing not only the gaps in surgical skills and confidence of MLs but also their knowledge and awareness of adverse events. One surgeon described a scenario where no adverse events had been reported by the hospital’s MLs, even though it was a requirement and he noticed that such events were occurring. The surgeon reported that rather than intentionally avoiding the documentation of adverse events, the MLs were not trained to identify and document them.

I think the issue here was the MLs understanding what adverse events are. There would be adverse events and they wouldn’t report them, not that they didn’t want to but because they didn’t know (SS 5)

### Obstacles and enablers to surgical supervision

Study participants reported that shortages of essential resources, both human and physical, at the DLH precluded supervision achieving maximum benefit; in that, specialists were unable to supervise and guide the MLs in the full range of procedures that could be performed at the district level, and at times, when supervisory visits were taking place, operations could not be undertaken at all.

The first factor limiting the potential of surgical supervision noted by interviewees was the insufficient number of skilled staff in district hospitals. Notably, the low number of theatre nurses and skilled anaesthesia providers prevented surgeons and MLs from performing some major surgeries. The majority of theatres had a single qualified anaesthetist, or in certain cases, none at all; this prevented the performance and supervision of any surgeries that required significant anaesthetic attention.

So even as I went around to do my supervisory visits, we could only do so much due to the skills shortage. You could find that there is only one nurse with anaesthetic skills, she can’t intubate, she can’t do everything so you end up doing not very major surgery (SS 4)

Secondly, the lack of functioning essential equipment was listed as a barrier to effective supervision, in respect to some procedures, and surgeons and MLs reported instances of cancelling procedures altogether due to the lack of anaesthetic monitors or other, more basic, equipment. One surgeon in particular refused to take the risk of starting an operation without the assurance of adequate postoperative monitoring.

Sometimes we say if you are not going to ‘do (*manage the patient*) postoperatively’, do not even start it. (SS 1)

Thirdly, a supervising surgeon reported concerns regarding electricity load shedding. Power would be repeatedly cut-off when operations were ongoing; in certain cases, it would not return for an entire day. This led to other obstacles, such as the inability to use the autoclave for sterilisation of surgical instruments which affected the overall effectiveness of the tested intervention.

## Discussion

This paper reports original in-depth findings on the feasibility of, and the actual benefits and obstacles to, implementing a surgical specialist supervision model as a means of improving surgical skills at DLHs in sub-Saharan Africa. The findings suggest that the model is an effective way of ensuring clinical supervision of remotely located mid-level surgical providers through a programme of regular visits by surgical specialists.

Surgical outreach campaigns that enable specialists to intermittently or occasionally deliver services to rural populations have served a similar purpose to the COST-Africa supervision model and have often resulted in good outcomes [25]. The main concern, however, is that such campaigns seldom incorporate training and supervision of the district-level staff who provide regular and accessible services to these communities, as well as their lack of sustainability [26]. To avoid this, COST-Africa introduced a combined training and supervision approach to make surgical expertise available in a sustainable way in Zambia’s rural communities, both through surgical specialists making regular quarterly supervisory visits and being available for phone consultations. The use of local surgical specialists, developing and strengthening their relationships with the surgical teams at the district hospitals that manage and refer patients to the specialist hospital, is one component of a sustainable, surgical quality of care intervention. Making regular surgical supervision sustainable was enhanced by the central involvement of the research principle investigator (JK) and two of the project’s surgical supervisors in the development of Zambia’s National Surgical, Anaesthesia and Obstetric Plan (NSOAP) [27]. The cornerstone of the national plan, building on a 10-year national programme of deploying surgically trained MLs to DLHs [10], is the tested programme of regular surgical supervision provided by the COST-Africa research [27].

In an earlier paper, we discuss the importance of a participatory implementation research approach that brings together local researchers, supported by external researchers, in informing the development of NSOAPs. This research team continues to work on refining and evaluating the supervisory model, under the follow-up SURG-Africa project [28] in close collaboration with the Ministry of Health in Zambia. This close collaboration, which started in 2011, increases the likelihood that the supervisory model will form part of a sustainable national programme. Modifications were made to the intervention based on the lessons learned from the supervision model tested in COST-Africa. Other specialists were included in in the supervisory visits (obstetricians, anaesthesiologists and OT nursing specialists) aiming to provide training and supervision for other DLH essential staff to the provision of safe surgical services. The focus is on the skills of individuals involved in surgery and how they work together (teamwork). This is one of the ways in breaking the barriers between MLs and MDs in particular, identified in this study, by making them work together under the supervision of specialists.

This paper also aims to contribute to a broader understanding of how to optimise supervisory models for NPCs working at DLHs in sub-Saharan Africa (SSA). Although similar initiatives have been implemented in other countries [25], to date, NPC supervision is still irregular or often lacking in rural health care systems [15, 29], and there is little evidence about the practical implementation of NPC supervision models. This study to our knowledge was the first one to explore these dimensions in practice. It provides evidence to illustrate the additional role that specialist surgeons can play, bringing the benefits of their expertise to rural populations, where such surgical competence would otherwise be unobtainable. Given the concerns that have been expressed, it adds to evidence [30] showing that the benefits of NPC-delivered surgery in rural areas outweigh the risks such as sub-optimal quality of surgery or ‘task creep’ [31], if effective surgical systems (including specialist supervision) are in place.

DLHs are at the frontline of the provision of surgical care for the majority of the population in Zambia and SSA, but often lack sufficient capacity, especially in staff surgical skills, to meet the needs of rural populations [32]. The study identified several direct benefits that ensued due to the supervision model, ranging from individual-level developments in the MLs’ surgical capabilities to wider institutional-level advancements experienced by the DLHs. On an individual clinician level, the supervision resulted in self-reported increased confidence and skills among the MLs which confirms the effectiveness of this method observed in other studies [33, 34]. Through the support of their assigned supervising surgeons, the majority of MLs expressed increased confidence in their surgical capabilities, as well as the ability to take on more complex procedures.

The surgeons also played a role in improving the working environment at intervention hospitals by strengthening relationships between MLs and MOs. It has previously been documented that in the district hospital setting, although medical doctors (MDs) have higher medical education than MLs, the consistent exposure of MLs to surgery while in training has led to their skills exceeding those of the doctors at times [26]. Tension revolving around seniority, role definition and the scope of decision-making/practice boundaries are some work environment issues in the relationship between the two cadres that have previously been reported in other studies [4, 6, 35]. Although most MOs in this study appreciated the MLs’ surgical capabilities, some surgeons reported they had resolved disputes between MLs and MOs which, if left unresolved, could have potentially affected the delivery of surgery or even compromised patient care.

The supervision also improved the functional dynamics of DLHs on an institutional level. By improving the individual-level skill sets of MLs and establishing communication channels between DLHs and central hospitals, the local surgical teams were able to undertake more cases [20] and avoid unnecessary referrals to the central hospitals. Interviewed cadres also reported the cost benefits of reducing such referrals, confirming findings from other studies with other studies carried out in the SSA region [35–38]. Additionally, these communication channels between the hospitals also contributed to the overall safety of surgical practice at rural hospitals.

The newly created communication channels proved to be life-saving during the management of complex cases, especially in situations where urgent intervention was required. Moreover, the interviewed surgeons reported the importance of periodically placing provincial surgeons in rural areas in terms of lobbying and advocating. Due to the severe shortage of consultant surgeons in Zambia [39], their influential power at a provincial level is yet to be utilised. Considering almost all cadres reporting the issue of limited surgical supplies, a frequently occurring phenomenon in the SSA region [40], the exposure of DLHs to provincial surgeons also appeared to be effective in that sense.

Although this intervention provided promising outcomes, the study identified several instances where surgeons were incapable of mentoring the MLs due to the unavailability of equipment, such as anaesthetic monitors, or key surgical staff. For example, since the effective management of surgical cases must include preoperative, intraoperative and postoperative care, supervising surgeons refused to carry out procedures if these aspects of patient care were to potentially be undermined. Not only do such barriers waste the surgeons’ invaluable visits and lead to missed opportunities, but also compromise the standard of surgical practice altogether.

Since MLs account for a very large proportion of the surgical workforce at DLHs [10], the concurrent inadequacy of developmental opportunities for these cadres leaves the quality and safety of their practice open to question [26]. In order to ensure ongoing clinical competency and ethically acceptable standards of care at DLHs, consistent schemes for NPC supervision and education must be integrated into a regulatory framework [26]. It is plausible that there were other system-wide effects, whereby regular visits by surgical specialists to DLHs benefitted other clinical services and hospital management more broadly, but such effects were not measured. The Zambia National Health Sector Strategic Plan 2011–2015 [19] called for the development of outreach programmes from technical supervision of regional referral hospitals by specialists at national tertiary hospitals; our findings suggest there is a scope for the supervision to be extended to the district level, focusing initially on essential surgery.

## Conclusions

The supervision model tested in COST-Africa was adopted locally and incorporated into the National Surgical, Anaesthesia and Obstetric Plan (NSOAP) which emphasises the need to use MLs and MDs in DLHs for the provision of surgical services in Zambia, and the need to supervise them regularly [27]. Further research is needed to establish the cost-effectiveness of the supervision model, to identify any opportunity costs in terms of surgical specialist spending periods of 1–3 days away from their specialist hospitals while providing supervision in districts (DLHs in Zambia are widely dispersed), and also to assess if the principles of participatory implementation research in the new SURG-Africa project are translated into long-term sustainable models for quality-assuring surgical services for the district and rural populations in Zambia.

## Limitations

Firstly, limitations include possible bias of the interviewees and the Hawthorne effect, they were all involved in a health system strengthening the COST-Africa project and the sample size was relatively small. Both supervisors and supervisees may have wanted to depict the intervention in a positive way. Secondly, although there was a self-reported overall rise in the output and scope of district-level surgery, the findings described in our previous publication assessing the effectiveness of the model through an RCT were not conclusive [20] for all hospitals taking part in the trial.

## Data Availability

The datasets generated and/or analysed during the current study are not publicly available due to its confidential nature and potential sensitivity issues resulting from data disclosure but are available from the corresponding author on reasonable request.

## Abbreviations

COST-Africa: Clinical Officer Surgical Training in Africa
C/S: Caesarean section
DLH: District-level hospital
DMO: District medical officer
ML: Medical licentiate
MD: Medical doctor
SS: Supervising surgeon
MoH: Ministry of Health
NPC: Non-physician clinician
NSOAP: National Surgical, Obstetric and Anaesthesia Plan
PMO: Provincial medical officer
RCT: Randomised control trial
SRQR: Standards for Reporting Qualitative Research
SSA: Sub-Saharan Africa
UTH: University Teaching Hospital
